# The impact of fine particulate pollution (PM2.5) on hospitalization costs in China

**DOI:** 10.3389/fpubh.2025.1683761

**Published:** 2025-12-03

**Authors:** Chen Chen, Chen Ma, Xingyue Wu, Jinglin Song, Juanjuan Yan

**Affiliations:** 1 School of Public Finance and Economics, Shanxi University of Finance and Economics, Taiyuan, China; 2 China Center for Special Economic Zone Research, Shenzhen University, Shenzhen, China; 3 School of Economics and Finance, Chongqing University of Science and Technology, Chongqing, China; 4 School of Health Services and Management, Shanxi University of Chinese Medicine, Jinzhong, China

**Keywords:** air pollution, hospitalization costs, land use types, generalized random forest, PM2.5

## Abstract

**Introduction:**

Air pollution poses a threat to public health and socio-economic stability, significantly increasing the disease burden on the population and causing heavy economic impacts, especially in terms of medical expenses. Quantifying this economic impact is crucial for formulating effective public health strategies. This study aims to deeply explore the direct impact of air pollution on specific medical expenses.

**Methods:**

This study utilized the medical data of inpatients in a certain city in southwest China from 2014 to June 2019, the data of key air pollution indicators such as PM2.5 and AQI collected through local monitoring stations, and the data of land use types in this city.

**Results:**

(1) Air pollution has significantly increased the total hospitalization costs for patients and is a key factor leading to the rise in their medical expenses.Taking the 7-day moving average as an example, a 10 μg/m³ increase in PM2.5 led to a 0.5% rise in total hospitalization costs, equivalent to about 42 yuan per individual. (2) Air pollution has significantly increased the amount of medical insurance reimbursement for patients rather than their out-of-pocket expenses, suggesting that patients tend to mitigate related expenses through insurance reimbursement. (3) Green space area can effectively alleviate the increasing effect of air pollution on hospitalization costs, while industrial land has the opposite effect.The mitigating effect of green spaces on air pollution is most prominent among middle-aged and older adults groups and is more significant under conditions of low wind speed and precipitation.

**Discussion:**

Air pollution has exerted economic pressure on both individual patients and the medical security system. The research results can provide important references for optimizing the allocation of medical resources and strengthening health protection to reduce the health and economic burden of air pollution.

## 1 Introduction

Air pollution control has been a global priority, especially for developing countries (1). In the past two decades, China’s rapid economic growth has led to environmental deterioration, with air pollution a major issue. The Global Burden of Disease 2019 study indicates that air pollution in China was responsible for 1.85 million deaths in 2019, with up to 1.42 million of these deaths directly related to particulate matter pollution. In 2019, only 157 out of 337 prefecture-level and above cities met national air quality standards (2). In response, the Chinese government has taken measures. In 2013, the State Council promulgated the Action Plan for the Prevention and Control of Air Pollution (APCP), which was implemented and resulted in a significant improvement in air quality, especially in the concentration of PM2.5 (3). Later, in 2018, the Central Committee of the Communist Party of China (CPC) and the State Council launched the Three-Year Action Plan for Winning the Battle for the Blue Sky (2018–2020) to mitigate the harm of pollutants like PM2.5 and PM10 to public health (4). However, research on the impact of improved environmental quality on population health expenditures remains scarce and merits further study.

In recent years, air pollution research has been advancing. Growing attention is given to assessing its health and economic impacts from environmental aspects. Deschenes et al. (5) found that air pollution had a significant impact on weight gain and obesity rates, which led to more than $270 million in healthcare expenditures. In contrast, Qin et al. (6) examined the impact of air pollution on housing transactions. Their findings indicated that during periods of elevated air pollution, homebuyers’ willingness to pay (WTP) is significantly higher, which in turn drives the price of housing transactions to increase by 0.65%. Furthermore, Li et al. (7) gauged the economic gains from air quality enhancement. By using insurance data from a large western Chinese city and applying the Grossman model with inverse temperature as a crucial variable, they found that for every 10 micrograms per cubic metre drop in PM2.5, average healthcare costs fall by $263.6 and man-hour losses due to health issues also diminish. These studies not only expose the extensive health and economic impacts of air pollution but also furnish a firm foundation for policymakers.

Existing literature on air pollution has predominantly concentrated on source apportionment and characterizing pollutant concentrations, with comparatively less emphasis on quantifying the subsequent economic burden on healthcare systems. Extensive epidemiological evidence has established that long-term exposure to PM2.5 increases the risk of cardiovascular diseases (1, 8, 9), respiratory diseases (10), lung cancer (11), and asthma (12). Acute exposure to air pollution is related to risks of accidents like traffic accidents (13) and infant mortality (14, 15). The underlying pathophysiological mechanisms, including the induction of oxidative stress, inflammatory responses, and DNA damage, further substantiate the direct impact of air pollution on human health (16). While these direct health effects are well-documented, research that translates these risks into quantified healthcare economic burdens remains scarce. This gap may lead to an underestimation of the social cost of air pollution and hinder effective public health resource allocation. The economic and health losses associated with air pollution are not solely reflected in direct healthcare costs. They also manifest in the increased self-protection costs that individuals must bear to safeguard themselves against air pollution, such as purchasing air purifiers (17), relocating their residences (18, 19), and procuring enhanced quantities of filtering masks and health insurance (20–22). It is important to acknowledge that while wearing a mask can mitigate the health risks associated with air pollution, it also entails a range of hidden costs, including discomfort and inconvenience (23). Additionally, studies have demonstrated that individuals are willing to spend more money to avoid such discomfort and inconvenience than the cost of purchasing a mask. The deterioration of air quality has been found to have a detrimental impact on both physical and mental health, as well as increasing healthcare costs. This poses a significant threat to public health and well-being.

When exploring environmental quality-public health links, land use patterns (a key human-nature link) affect air pollution disease burden. Urban land uses like industrial land and green spaces alter pollutant processes, influencing health risks and disease burden. Industrial land concentration boosts pollutants and respiratory diseases; green spaces alleviate them. Analyzing this mechanism helps formulate targeted policies. Optimizing land use can reduce air pollution health hazards, supporting public health. The research on the relationship between land use types and air pollution is of great significance. Some studies employed land use regression models to explore the relationship between PM2.5 and land use in major cities in South Korea. It was found that the PM concentrations and variability in commercial areas, business districts, industrial areas, mixed areas, and high-density residential areas showed statistically significant differences compared with parks and green Spaces (24); regarding the research on Seoul, South Korea, some scholars have also pointed out that industrial land will increase PM levels in all cases (25). Another part of the studies explored the spatio-temporal evolution of PM2.5 exposure through spatial autocorrelation analysis and found that the annual PM2.5 exposure in construction land was the highest, while that in forests was the lowest (26). Another part takes Nanchang City, China as the research object. The research shows that land use has a significant impact on PM2.5 levels, and urban functional zones are the appropriate spatial scale for studying the influence of land use types on PM2.5 pollution in urban areas. Traffic conditions are the main influencing factor of PM2.5 pollution in Nanchang urban area (27). Furthermore, changes in land cover are also a key factor influencing changes in air pollution. Changes in land cover types can lead to alterations in the sources and sinks of air pollutants, thereby affecting the spatial distribution of PM10 (28).

This paper examines the cumulative effect of fine particulate matter (PM2.5) on individual hospitalization costs. Using 2014–2019 hospitalization costs data of patients with respiratory and cardiovascular diseases in a southwestern Chinese city. The results show that air pollution significantly increases the total hospitalization costs, and the effect intensifies with the increase of cumulative exposure duration. The impact of air pollution on hospitalization costs is regulated by different land use types. Green space land significantly mitigates the enhancing effect of air pollution on hospitalization costs. Industrial land significantly amplifies the enhancing effect of air pollution on hospitalization costs. Moreover, the study explores the nonlinear heterogeneity influence of green space regulation effects based on the generalized random forest algorithm.

This paper makes significant contributions. First, it uses a large and detailed dataset of inpatient medical records from a southwestern Chinese city (2014–2019.6), including cost, diagnosis, reimbursement, and institution details, strongly supporting the analysis of air pollution and healthcare burden. In terms of research areas, although the relationship between air pollution and patients’ healthcare costs has been explored (32, 33), most studies have focused on the level of health insurance reimbursement costs, this paper comprehensively analyses total hospitalization expenditure, out-of-pocket payments, and reimbursement. Second, this paper incorporates the moderating effect of land use patterns to explore the moderating role of green Spaces and industrial land on the impact of air pollution. Finally, in this paper, the causal forest algorithm in the generalized random forest algorithm is used to explore the nonlinear heterogeneous influence of the moderating effect and analyze its changes with factors such as the patient’s age, regional wind speed, and regional precipitation. This study provides policy references for the rational utilization of land planning to alleviate the negative impacts of air pollution.

## 2 Materials and methods

### 2.1 Study area

The study city lies in the inland Yangtze River’s middle and upper reaches in China. In 2023, it had a population of 32 million and an urbanization rate of 71%. It has a humid subtropical monsoon climate. Its per capita GDP is high in China, and with a large population, there are rich healthcare resources. There are 21,000 healthcare institutions, including 860 hospitals and 20,000 primary ones. The number of beds per 1,000 people is 6.4. Thus, it can be a representative case. The annual average PM2.5 concentration in the city we studied is approximately 49 μg/m^3^. This range highly represents the air pollution challenges faced by many mid-latitude inland cities in China. Due to industrial activities and specific topographic conditions, these cities often experience moderate to high pollution levels.

### 2.2 Air quality and temperature data

To understand the air quality in sample cities, data on key air pollution indicators like PM2.5 and AQI were collected from 2014 to 2019.6 from local monitoring stations. The summary statistics are shown in Table 1. Table 1 shows that the average PM2.5 concentration was 49 μg/m^3^, PM2.5 exceeded the National Secondary Concentration Limit, as compared to the Chinese Ambient Air Quality Standards. Meteorological data such as wind speed and precipitation were also collected to reflect daily weather conditions considering their potential influence., This article refers to relevant literature (7), the core independent variable is the air pollution level, which is measured by the moving averages of PM2.5 over the 3 days and 7 days prior to the patients’ admission (all the aforementioned days include the day of admission). The impact of PM2.5 on human health has a cumulative effect. Short-term exposure is prone to trigger acute physiological responses in the respiratory and cardiovascular systems, thereby increasing the demand for hospitalization. The 3-day average value can effectively capture the immediate impact of short-term PM2.5 exposure on the hospitalization rate. Choosing the 7-day average value can more stably reflect the cumulative effect of pollution exposure. To control the potential impact of climate factors, the climatic-level control variables include wind speed and precipitation.

**Table 1:** Description and summary statistics of key variables.

Variable names		Variables	Mean	Std. Dev	Min	Max	Unit
Dependent variable		LnTotal costs	8.52	1.01	0.00	14.02	Yuan	
		LnReimbursemen costs	7.96	2.01	0.00	14.00	Yuan	
		LnOut-of-pocket costs	5.06	2.69	0.00	13.72	Yuan	
Independent variable		3-day average Pm_2.5_	48.67	28.30	8.50	179.25	μg/m^3^	
		7-day average Pm_2.5_	48.56	26.37	8.50	176.25	μg/m^3^	
		AQI	72.82	37.05	14.00	262.00	–	
Control variables	Climatic-level control variables	Wind speed	1.33	0.39	0.30	3.70	m/s	
		Precipitation	3.55	9.23	0.00	111.20	mm	
	Individual-level control variables	Gender	0.55	0.50	0.00	1.00	–	
		Age	50.44	30.01	0.00	104.00	Years old	
		Length of stay	8.50	10.43	0.00	773.00	day	
		Plan for re-hospitalization	0.04	0.19	0.00	1.00	–	
		Hospital grade	2.11	0.90	0.00	3.00	Grade	
Moderating variable		LnGreen	6.13	2.42	0.00	8.86	Ten thousand mu	
		LnIndustrial	3.27	1.82	0.04	5.55	Ten thousand mu

*The unit of the variable is the unit before taking the logarithm. Variables marked with “ln” in the symbol are all processed by taking the logarithm.

### 2.3 Medical data

The dataset encompasses daily hospitalization records of sample cities from 2014 to 2019. It includes detailed cost data, such as total, out-of-pocket, reimbursement, and drug costs, as well as patient information like disease type, age, gender, and hospital details. Data was screened by ICD-10 codes for cardiovascular (I00-I99) and respiratory (J00-J99) diseases and then matched with air pollution data. The data-matching procedure uses each patient’s admission date from the hospitalization database as the temporal reference. The moving averages of PM2.5 over the 3 days and 7 days preceding (and including) the admission date are employed as the air pollution indicators, serving as the core explanatory variables. The total hospitalization cost for each patient is treated as the dependent variable. Other environmental variables, including air quality and meteorological data, are matched using the same approach. The mean total hospitalization cost was 8,330 Yuan, with 1,380 Yuan out-of-pocket and 6,950 Yuan reimbursed. To ensure better analysis, these costs were logarithmically transformed. The summary statistics are shown in Table 1. All the data used in this study were taken from the China Regional Health Information Platform, the research strictly adhered to the following protocols throughout the investigation: (1) Data collection completely avoids personally identifiable information; (2) only objective indicators that meet policy requirements are standardized; (3) statistical analysis conducted exclusively at the population-level mean. All research procedures in this paper were approved by the Regional National Health Committee of China. The dependent variables are total costs, reimbursement costs, and out-of-pocket costs. The individual-level control variables include patients’ age, gender, length of hospital stay, whether there is a plan for re-hospitalization within 31 days after discharge, and hospital level.

### 2.4 Land use data

The land use data in this paper is based on the “Classification of Current Land Use Status” (GB/T 21010-2017) standard, and is obtained by integrating the data from the second and third national land surveys and the annual land change survey. The national standard “Classification of Current Land Use Status” adopts a two-level classification system of first and second levels. The first level includes 12 major categories. The land survey data lacks the data of 2017 and 2018. Therefore, the land use data of this study covers four major first-level categories of forest land, grassland, urban construction land and mining land in each district (county) of this city from 2014 to 2016 and 2019, and is matched with the data of the district (county) where the patient currently resides. The absence of land use data for 2017 and 2018 only affected analyses that incorporated land use as a moderating variable. For all other parts of the empirical analysis, including the baseline regressions and robustness checks, we used the complete and uninterrupted dataset spanning from 2014 to June 2019. The green space area is measured by the sum of forest land area and grassland area, while the industrial land area is measured by the sum of urban construction land area and mining land area. The summary statistics are shown in Table 1.

### 2.5 Model

Our baseline regression equation is shown in Equation 1, where footnote denotes the individual and denotes the admission time. Includes individual hospitalization expenditures, which are divided into total costs, reimbursement costs, and out-of-pocket costs and are all logarithmic in nature. Refers to the air pollution level to assess the effect of exposure to air pollution of different lengths of time on hospitalization expenditures, this paper measures the air pollution level using the moving averages of PM2.5 over the 3 days and 7 days before hospitalization (the day of hospitalization is included in both periods) indicates the Climatic-level control variables and the individual-level control variables.


$$\ln\mathrm{Cos}t_{\mathrm{it}}=\beta_{0}+\beta_{1}\text{Pollutio}n_{\mathrm{it}}+\beta_{2}\text{Contro}l_{\mathrm{it}}+\gamma_{v}+\delta_{d}+\theta_{p}+\varepsilon_{\mathrm{it}}$$ (1)

In addition, represents the year fixed effects, represents the admission department fixed effects, represents the payment method fixed effects. Represents that the standard errors in this paper are clustered at the level of patients’ current residential addresses.

## 3 Results

### 3.1 Baseline results

Table 2 reports the effect of PM2.5 moving in the past for 3 and 7 days on total costs, reimbursements costs and out-of-pocket costs. Total costs equal out-of-pocket plus reimbursements. As shown in columns (1) to (2) of Table 2, for total costs, PM2.5 had a significant positive effect. The coefficient increased with the moving average. Take the 7-day moving average as an example, a 10 μg/m^3^ increase in PM2.5 led to a 0.5% rise in total costs, about 42 yuan per individual, showing fine particulate matter’s cumulative harm. For reimbursement costs. As shown in columns (3) of Table 2, the impact grew significantly after 7-day moving. Regarding out-of-pocket costs, as shown in columns (4) of Table 2, contrary to some studies (7, 29), we found that it is not significantly associated with the 15-day moving average. This could be due to people favoring insurance reimbursement to cut spending.

**Table 2:** Impact of PM_2.5_ on total costs, reimbursements costs and out-of-pocket costs.

Variables	(1)	(2)	(3)	(4)
	LnTotal	LnTotal	LnReimbursement	LnOut-of-pocket
3-day average Pm_2.5_	0.0004***	0.0002**	0.0002	−0.0005
	(0.0001)	(0.0001)	(0.0001)	(0.0006)
Control	No	Yes	Yes	Yes
Fixed effects	Yes	Yes	Yes	Yes
Observations R-squared	380,385	330,956	330,956	330,956
	0.227	0.399	0.355	0.338
7-day average Pm_2.5_	0.0005***	0.0003***	0.0003**	−0.0006
	(0.0001)	(0.0001)	(0.0001)	(0.0006)
control	No	Yes	Yes	Yes
Fixed effects	Yes	Yes	Yes	Yes
Observations R-squared	380,385	330,956	330,956	330,956
	0.227	0.399	0.355	0.338

Variables	(5)	(6)	(7)	(8)
	10-day average Pm2.5	15-day average Pm2.5	7-day average AQI	10-day average AQI
Pollution	0.0003***	0.0004***	0.0002**	0.0002***
	(0.0001)	(0.0001)	(0.0001)	(0.0001)
Control	Yes	Yes	Yes	Yes
Fixed effects	Yes	Yes	Yes	Yes
Observations	330,956	330,956	330,956	330,956
R-squared	0.399	0.399	0.399	0.399

Variables	(9)	(10)	(11)	(12)
	Lassocv		Gradboost	
	3-fold	5-fold	3-fold	5-fold
7-day average Pm_2.5_	0.0003***	0.0002*	0.0003***	0.0002*
	(0.0001)	(0.0001)	(0.0001)	(0.0001)
Control	Yes	Yes	Yes	Yes
Fixed effects	Yes	Yes	Yes	Yes
Observations	330,961	330,961	330,961	330,961

*Numbers in parentheses indicate cluster standard errors. ***p < 0.01, **p < 0.05, *p < 0.1. The following are the same for below table.

### 3.2 Robustness tests

#### 3.2.1 Replace the independent variables

The study conducts a robustness test by replacing the independent variables to verify the stability of the benchmark regression results. The study measures the air pollution level using the 10-day moving average of PM2.5, the 15-day moving average of PM2.5, the 7-day moving average of AQI, and the 10-day moving average of AQI, respectively. The robustness of the baseline regression results can be verified through the regression results in Table 2.

#### 3.2.2 Testing of the double machine learning model

The concept of double machine learning was formally proposed by Chernozhukov et al. (34). Its core idea is to decompose the causal inference problem into two independent prediction steps and use machine learning algorithms to improve the accuracy and robustness of causal effect estimation. In this study, two machine learning methods, namely lasso regression and gradient boosting, are adopted. The first step is to fit the relationship between the dependent variable and the control variables to obtain the predicted residuals. The second step is to use the same methods to fit the relationship between the independent variable and the control variables to obtain the predicted residuals, with the parts explained by the control variables removed in both cases. The third step is to estimate the causal effect of the independent variable on the dependent variable by conducting a regression analysis on these two residuals. In this paper, 3-fold and 5-fold cross-validation are used, respectively. The dependent variable is the total cost, and the independent variable is selected as the 7-day moving average. The regression results are shown in columns (9)–(12) of Table 2, which verify the robustness of the regression results.

### 3.3 Moderating effect analysis

Land use mode refers to the total of various development, utilization, protection and improvement activities carried out by humans on land resources based on the natural attributes of the land and the demands of social and economic development. It includes various specific types such as forest land, grassland, wetland, urban construction land and industrial and mining land, and transportation land. In the process of air pollution affecting medical costs, different land use types play differentiated moderating roles. This paper mainly explores the role of green space and industrial land in the effect of air pollution on hospitalization expenditures. The survey scope does not include the data of 2017 and 2018. Therefore, this paper excludes the samples of these 2 years and conducts a moderating effect analysis.

#### 3.3.1 Green space land

The study measures the green space area at the county level by the total area of forest land and grassland in each district and county, the model is shown in Equation 2:


$$\begin{array}{l} \ln\mathrm{Cos}t_{\text{itcv}}=\beta_{0}+\beta_{1}\text{Pollutio}n_{\mathrm{it}}*\text{Gree}n_{\mathrm{ivc}}+\beta_{2}\text{Contro}l_{\mathrm{it}}\\ +\gamma_{v}+\delta_{d}+\theta_{p}+\varepsilon_{\mathrm{it}} \end{array}$$ (2)

In Equation 2, is the green space area in the district (county) c where patient’s current residence is located in year, the year when the patient is admitted to the hospital, it is measured by the sum of forest land and grassland areas. Due to data availability constraints, the data on green space area for District (County) c in this paper, covering the period from 2014 to 2016, were derived by multiplying the proportion of District (County) c’s green space area in 2019 relative to the total green space area of the urban district (county-wide total) by the total green space area of the urban district (county-wide total) in year v. The calculation method for district (county) level data of industrial land is the same as above. Represents the interaction term between air pollution level and green space area. The remaining variables are the same as in Equation 1. The results are shown in columns (1) to (2) of Table 3.

**Table 3:** Impact of interaction term between air pollution level and land area on total costs.

Variables	(1)	(2)	(3)	(4)
	LnTotal costs			
	Full sample		HS group	LS group
3-day average Pm_2.5_*Green	−0.0001***	−0.0002***	−0.00003	−0.0001***
	(0.0000)	(0.0000)	(0.00008)	(0.0000)
3-day average Pm_2.5_	0.0007**	0.0010***	0.0004	0.0003
	(0.0003)	(0.0003)	(0.0004)	(0.0003)
Green	0.0081	0.0149**	0.0086	0.0162
	(0.0066)	(0.0062)	(0.0212)	(0.0105)
Control	No	Yes	Yes	Yes
Fixed effects	Yes	Yes	Yes	Yes
Observations R-squared	188,802	155,928	61,317	94,609
	0.218	0.363	0.485	0.330
7-day average Pm_2.5_*Green	−0.0001***	−0.0002***	−0.00003	−0.0001***
	(0.0000)	(0.0001)	(0.0001)	(0.0001)
7-day average Pm_2.5_	0.0010***	0.0013***	0.0005	0.0006
	(0.0003)	(0.0004)	(0.0005)	(0.0004)
Green	0.0104	0.0174**	0.0090	0.0184*
	(0.0069)	(0.0066)	(0.0221)	(0.0104)
Control	No	Yes	Yes	Yes
Fixed effects	Yes	Yes	Yes	Yes
Observations R-squared	189,288	156,407	61,440	94,965
	0.218	0.363	0.485	0.330

Variables	(5)	(6)	(7)	(8)
	LnTotal costs			
	3-day average Pm_2.5_		7-day average Pm_2.5_	
Pollution*Industrial	0.0001***	0.0002***	0.0001***	0.0002***
	(0.0000)	(0.0001)	(0.0000)	(0.0001)
Pollution	0.0007***	0.0007**	0.0008***	0.0008**
	(0.0002)	(0.0003)	(0.0002)	(0.0004)
Industrial	−0.0045	−0.0090	−0.0053	−0.0097
	(0.0057)	(0.0063)	(0.0059)	(0.0067)
Control	No	Yes	No	Yes
Fixed effects	Yes	Yes	Yes	Yes
Observations R-squared	195,810	162,179	195,810	162,179
	0.218	0.362	0.218	0.362

The results show that the coefficient is significantly negative, indicating that green spaces can effectively alleviate the impact of air pollution on total costs. The reasons are as follows: First, green spaces can reduce the concentration of air pollution through physical and chemical processes. They can absorb particulate matter in the air and, through photosynthesis, absorb carbon dioxide and release oxygen, thereby reducing the total amount of pollutants that people are exposed to and directly lowering the incidence of respiratory diseases and other air pollution-related diseases. Second, green spaces can regulate local temperature and humidity, alleviating the urban heat island effect. The cooling and moisture-retaining effects of green spaces can reduce the intensity of pollutants’ stimulation to the human body. Moreover, green spaces provide a more stable living environment for microorganisms, and some beneficial microorganisms can inhibit the reproduction of airborne pathogens, reducing the probability of respiratory infections. Third, green spaces promote physical and mental health and enhance the human body’s resistance to pollution. Green spaces offer residents spaces for leisure and exercise. Regular outdoor activities can improve heart and lung function and enhance immunity, thereby increasing the body’s tolerance to air pollution.

Furthermore, the samples were divided into groups with a high proportion of secondary industry and a group with a low proportion of secondary industry. The results are shown in columns (3) to (4) of Table 3. Only in the group with a low proportion of secondary industry, the coefficient was significantly negative. This indicates that green Spaces can play a significant role in alleviating the increase in medical costs caused by air pollution in regions where the transformation and upgrading of the industrial structure is smooth, while their effect is limited in regions where the transformation and upgrading of the industrial structure is difficult. The reasons are as follows: First, in regions with a low proportion of secondary industries, there are fewer industrial activities, and the sources of pollution are relatively scattered, with simpler pollutant components, making the purification capacity of green Spaces more evident. Second, in regions with a high proportion of secondary industries, industries are dense and will emit a large amount of complex pollutants. The purification capacity of green Spaces has a certain limit. The speed at which they adsorb and decompose pollutants may not keep up with the speed of pollution emissions and thus cannot significantly alleviate the increase in medical costs caused by pollution.

#### 3.3.2 Industrial land

To further verify the above conclusions, we focus on the effect of the increase in the relative area of industrial land on the impact of air pollution on hospitalization Costs. Industrial land area is measured by the sum of urban construction land and mining land areas. The model is shown in Equation 3:


$$\begin{array}{l} \ln\mathrm{Cos}t_{\text{itcv}}=\beta_{0}+\beta_{1}\text{Pollutio}n_{\mathrm{it}}*\text{Industria}l_{\mathrm{ivc}}+\beta_{2}\text{Contro}l_{\mathrm{it}}\\ +\gamma_{v}+\delta_{d}+\theta_{p}+\varepsilon_{\mathrm{it}} \end{array}$$ (3)

In Equation 3, is the relative industrial land area in the district (county) c where patient’s current residence is located in year, the year when the patient is admitted to the hospital. The relative industrial land area is the difference between industrial land area and the green space area. Represents the interaction term between air pollution level and relative industrial land area. The remaining variables are the same as in Equation 1. The results are shown in columns (5) to (8) of Table 3.

The results show that the coefficient is significantly positive. The results show that the increase in relative industrial land will enhance the promoting effect of air pollution on total costs. The core reason lies in that the expansion of industrial land will amplify the negative impact of air pollution from both the source of pollution and the buffer of pollution control. From the perspective of pollution sources, industrial land use implies an expansion of industrial production scale, an increase in fossil fuel consumption, etc., directly pushing up regional air pollution concentrations and exacerbating the incidence of respiratory diseases and other conditions among residents. From the perspective of governance buffering, there is a spatial occupation relationship between industrial land and green space land. An increase in the area of industrial land may compress the layout green space land and weaken their ecological functions of adsorbing pollutants and purifying the air.

### 3.4 Further analysis: nonlinear heterogeneous effects

The analysis of the above moderating effect is based on the assumption of the traditional linear model that the policy effect changes linearly. In fact, the moderating effect of green space may be affected by various factors and exhibit significant nonlinear characteristics. Therefore, effectively identifying this nonlinear impact is crucial for scientifically evaluating the effect of the land use policy. The causal forest algorithm in the generalized random forest algorithm can construct a tree structure through recursive data partitioning, accurately stratify samples, obtain conditional average treatment effects, and conduct an in-depth analysis of how the moderating effect of green space dynamically evolves with factors such as patients’ age, wind speed, and precipitation. Referring to relevant studies (30, 35), this paper uses the “Causal Forests” command in the generalized random forest model to estimate the conditional average treatment effect (CATE). The treatment variable W, outcome variable Y, and feature variable X are consistent with the independent variables, dependent variables, and control variables in Equation 1. The fixed effect is the same as Equation 1. As the number of decision trees increases, the average processing effect of the moderating effect remains stable, indicating that the number of selected base decision trees can meet the accuracy requirements. The study focuses on exploring the nonlinear heterogeneous impacts based on the moderating effect of green space.

#### 3.4.1 Age

**Figure 1:**
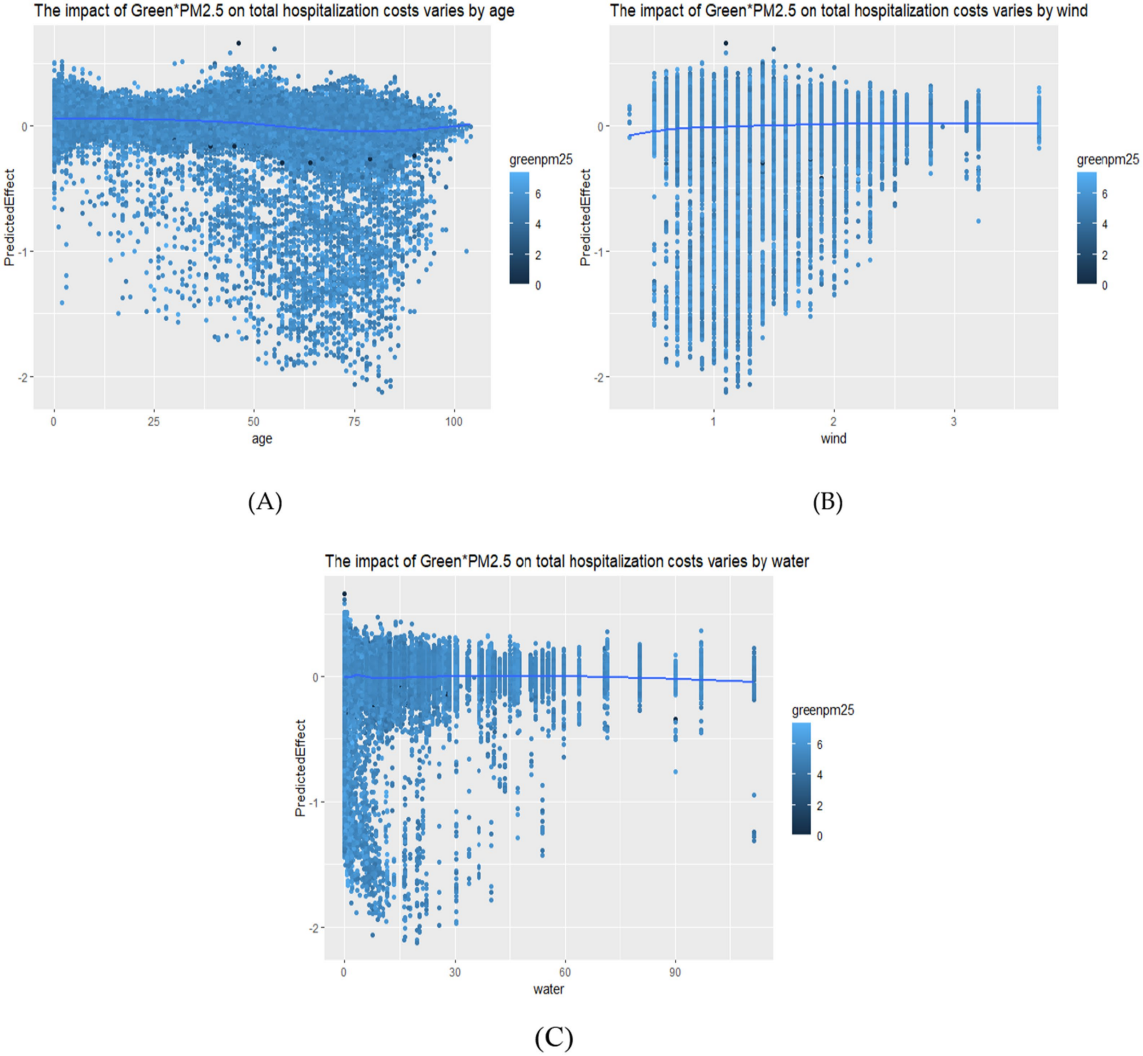
The nonlinear heterogeneous effect. **(A)** The effect by age; **(B)** The effect by wind speed; **(C)** The effect by wind precipitation.

The results are shown in Figure 1A, where the horizontal axis represents the variable of patients’ age and the vertical axis represents the impact of on total costs. The results indicate that the mitigating effect of green space is relatively gentle with no significant changes before residents reach 38 years old; after 38 years old, the mitigating effect strengthens. For the older adults aged between 65 and 85, the mitigating effect of green space on the impact of air pollution is the strongest. This may be because young people have a stronger ability to regulate against pollution, while the older adults have a weaker ability. Moreover, the same health damage leads to more complex complications and longer hospital stays for the older adults. Therefore, the additional protective effect of green space on young people is limited, but its protective effect on the older adults is significant.

#### 3.4.2 Wind speed

The results are shown in Figure 1B, where the horizontal axis represents the variable of wind speed and the vertical axis represents the impact of on total costs. When the wind speed is low, the mitigating effect of green space is significant, but it is not obvious when the wind speed is high. At low wind speeds, air pollutants stay near the ground for a longer time, and the leaves and branches of green space can effectively intercept and adsorb particulate matter, with the retention time being sufficient for green space to adsorb them. High wind speeds accelerate the diffusion of pollutants, making the retention time insufficient for green space to adsorb them. Additionally, they cause the settled air pollutants to be resuspended. Meanwhile, strong winds accelerate evaporation, reduce the humidity in the surrounding areas, and weaken the ability of plants to adsorb particulate matter through transpiration.

#### 3.4.3 Precipitation

The results are shown in Figure 1C, where the horizontal axis represents the variable of precipitation and the vertical axis represents the impact of on total costs. In the low precipitation scenario, the role of green spaces is more significant, while in the high precipitation scenario, the role of green spaces is not significant. In the case of low precipitation, wet deposition in the atmosphere is weak, and air pollutants such as PM2.5 rely more on the ecological purification functions of green spaces to reduce their concentrations—for example, through leaf adsorption, micro-environmental circulation driven by plant transpiration, and other processes. In the case of high precipitation, strong wet deposition itself becomes the dominant force in removing air pollutants. Abundant precipitation can directly remove particulate matter such as PM2.5 from the atmosphere through scouring, significantly reducing air pollution concentrations. Under such circumstances, the purification function of green spaces is overshadowed by the powerful pollutant removal effect of precipitation, and their additional effect on reducing pollutant concentrations is limited. Meanwhile, high precipitation may indirectly impair the ecological functions of green spaces. Excessive precipitation can lead to soil water logging in green spaces, affecting plant root activity and leaf physiological status, thereby reducing the efficiency of pollutant adsorption. Alternatively, precipitation may keep the surface of plant leaves continuously moist, which instead reduces their capacity to retain particulate matter.

## 4 Discussion

This study quantifies the impact of air pollution (PM2.5) on residential hospitalization costs using inpatient data from a southern Chinese city (2014–2019.06). Quantifying this economic impact is crucial for formulating effective public health strategies.

First, our result that air pollution significantly increases hospitalization costs. Taking the 7-day moving average as an example, a 10 μg/m^3^ increase in PM2.5 led to a 0.5% rise in total hospitalization costs, equivalent to approximately 42 yuan per person. Builds on prior research linking air pollution to healthcare expenditures. Barwick et al. estimated that reducing PM2.5 in China would lower national healthcare spending (31), and Zheng et al. highlighted the association between particulate matter and respiratory disease costs (29). Our study narrows this focus to hospitalization costs, a critical component of healthcare expenditure, and further identifies reimbursed costs as the primary driver. This specificity underscores the role of public healthcare systems in bearing the economic burden of air pollution, suggesting that air pollution not only harms individual health but also imposes fiscal pressures on social security frameworks—an aspect less emphasized in earlier work.

Second, the mitigating effect of green space on air pollution-induced hospitalization costs resonates with literature documenting green space’s role in reducing air pollution (24, 26). Our empirical findings reveal that green space can also alleviate the economic burden caused by air pollution, specifically by reducing hospitalization costs. Kim and Xu et al. found that industrial land aggravates air pollution (25, 28). Based on a nonlinear analysis using the Generalized Random Forest (GRF) method, we found that green spaces exhibit a context-dependent mitigation effect on health burdens. This protective role is strongest among the older adults (65–85 years) and becomes particularly crucial under calm weather conditions (low wind speed and low precipitation), when pollutants tend to accumulate. This suggests that the health benefits of green spaces are not uniform but play a compensatory protective role when exposure risks are highest. The finding provides an important scientific foundation for implementing precision-based urban greening strategies.

Based on the above research results, this study puts forward the following suggestions: (1) Establish a group health early warning and intervention mechanism based on air quality. When the PM2.5 concentration exceeds the standard, protection guidelines will be pushed to residents through community grids, medical institutions and media platforms. Community health service centers will provide free basic protective supplies and open emergency green channels for patients with chronic respiratory/cardiovascular diseases. (2) Optimize the allocation of medical resources to address the burden of pollution-related diseases. Dynamically adjust departmental resources. During the high-pollution season, increase the reserves of medicines and medical staff in relevant departments in advance. Promote remote diagnosis and treatment, implement remote monitoring for patients with pollution-sensitive diseases, and provide early intervention through family doctor teams to prevent the deterioration of the condition and hospitalization.

Expanding the research scope to a broader geographical area would enhance the generalizability of the research conclusions. In conclusion, this study quantified the economic burden that air pollution imposes on the public health system and identified the land use factors that regulate this economic cost. These insights offer practical approaches to mitigating the social and financial impacts of air pollution through targeted health intervention measures.

This study also opens promising avenues for future research. While our findings are robust and offer valuable insights for cities with comparable levels of air pollution, socioeconomic development, and health insurance systems, their generalizability to regions with markedly different climatic conditions, economic structures, or pollution control histories may be limited and warrants further validation. Future research could extend this analysis to the national level by integrating air pollution exposure monitoring, longitudinal health follow-up data, and comprehensive medical expenditure records across multiple regions. Such efforts would facilitate more rigorous cross-regional comparisons and strengthen the external validity of our conclusions.

## Data Availability

The data analyzed in this study is subject to the following licenses/restrictions: The medical data used in this study are available from China Regional Health Information Center but restrictions apply to the availability of these data, which were used under license for the current study, and so are not publicly available. Data are however available from the authors upon reasonable request and with permission of China Regional Health Information Center. Air pollution data are obtained: https://sthjj.cq.gov.cn/hjzl_249/. Meteorology data available from: https://data.cma.cn/. Land use data available from: https://gtdc.mnr.gov.cn/Share#. Requests to access these datasets should be directed to https://sthjj.cq.gov.cn/hjzl_249/; https://gtdc.mnr.gov.cn/Share#/.
